# Supplementary material for: Full-field structured-illumination super-resolution X-ray transmission microscopy
Source: Nat Commun. 2019 Jun 7;10:2494. doi: 10.1038/s41467-019-10537-x (PMC6555788; doi:10.1038/s41467-019-10537-x)
Supplement: Supplementary file 1 — Supplementary Information Final [file 41467_2019_10537_MOESM1_ESM.pdf]

Full-Field Structured-Illumination Super-Resolution X-ray Transmission Microscopy

B. Günther *et al.*

Supplementary Information

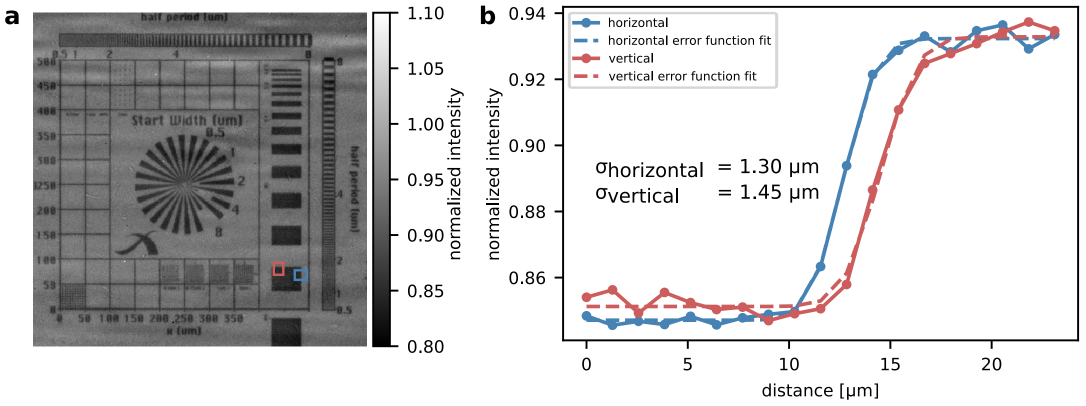

Supplementary Figure 1: Determination of the detector resolution. **a** reference corrected image of the test pattern acquired with a standard parallel beam. **b** Edge profiles along the vertical (red) and horizontal (blue) detector direction together with the corresponding fits. Resolution in the horizontal direction (1.30 µm) is slightly better than in the vertical one (1.45 µm).

Supplementary Note 1: Detector resolution

Supplementary Figure 1 displays a reference image of the resolution pattern acquired with a standard parallel beam imaging geometry. The X-ray energy was set to 35 keV, the effective pixel size in this measurement was 0.64 µm and a 50 µm LuAG-scintillator was used as well. 100 of each, dark frames, reference frames and projections of the resolution pattern have been taken at an acquisition time of 1000 ms. In order to be consistent with the experiment, 2×2 binning has been performed for all images. Dark frames are subtracted from all reference images as well as projections in a first step. In a second step, a standard reference correction is performed dividing the projection by the reference frame. As the X-ray beam was unstable during data acquisition, this step can result in images containing strong artifacts, if the intensity fringes of the X-ray beam are shifted in the projection relative to the reference. In order to find the best combination of projections and reference with lowest artifacts in the region of the edge-

measurements, we performed the following procedure: First, reference correction for each projection was performed separately with each of the references. The corrected images with the lowest global image variance were selected in a second step. Among those, the edge fit was performed on the one with the best reference correction in the area of the edge scans, which was selected by the following criteria: 1) Minimal intensity difference between the background area of the region of interest for the horizontal and vertical edge measurement. 2) Lowest intensity variance in vertical direction, which was determined in a background area to the left of the square and parallel to the region of interest for the vertical edge measurement. The regions of interest for the two scans are displayed by red and blue boxes in Supplementary Figure 1a. Detector lines perpendicular to the edge have been averaged prior to the error-function fit to the respective edge. In the vertical direction, i.e. the horizontal edge, the detector resolution is 1.45 µm (r.m.s.), whereas in the horizontal one, it is 1.30 µm (r.m.s.). The corresponding error-function fits and line plots are depicted in Supplementary Figure 1b.

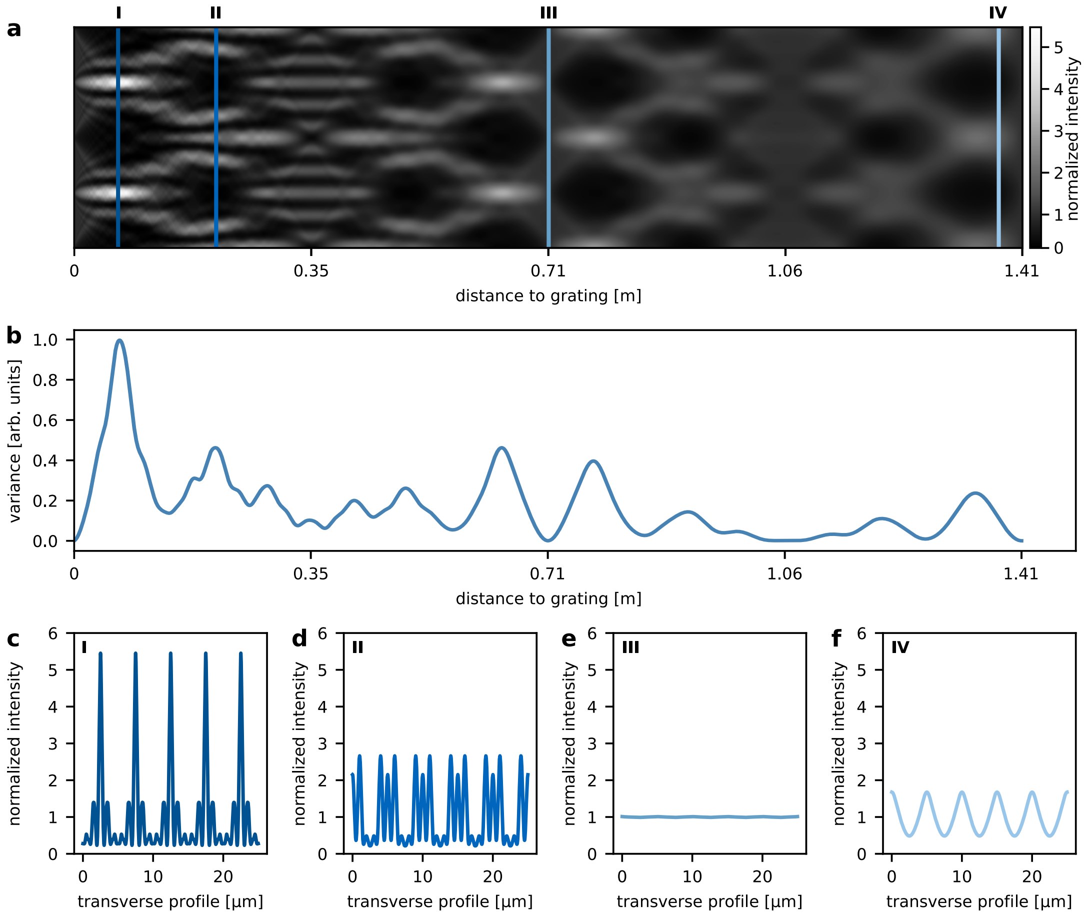

Supplementary Figure 2: Simulation of the Talbot-carpet with penumbral blurring. The Talbot-carpet of a triangular grating with a period of 5 µm and a height of 32 µm is simulated at an X-ray energy of 35 keV including X-ray absorption, the penumbral blur caused by the finite X-ray source size as well as the effect of the grating bridges. A horizontal source size of 36 µm given in [1] has been used in the simulation. **a** One full Talbot-distance of the Talbot-carpet is depicted for two simulated grating periods. **b** Variance perpendicular to the propagation direction. **c**-**f** show transverse intensity profiles at the same location within the Talbot-carpet as Figure 1. At the position of the experiment (**c**), the source blur is 27 nm and consequently barely effects the generated illuminations (0.7 µm).

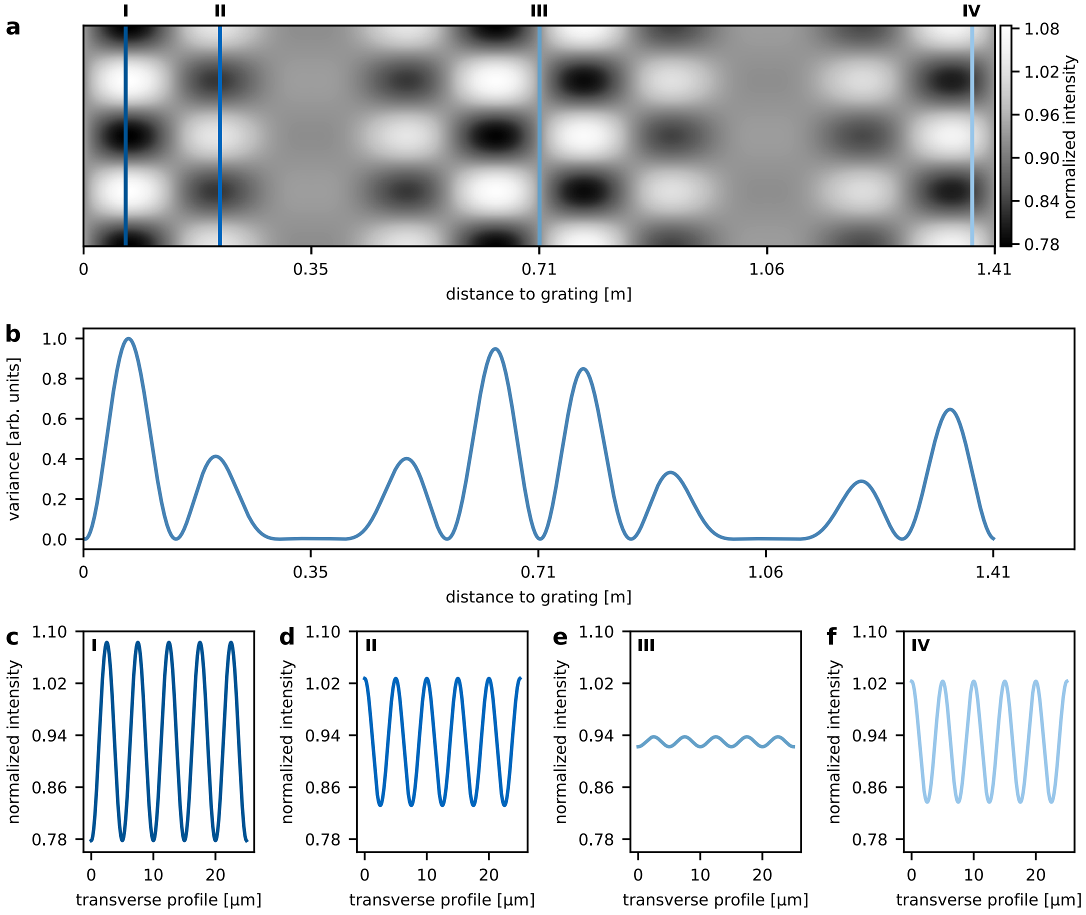

Supplementary Figure 3: Simulation of the Talbot-carpet with penumbral blurring, grating bridges and detector PSF. The Talbot-carpet of a triangular grating with a period of 5 µm and a height of 32 µm is simulated at an X-ray energy of 35 keV including X-ray absorption, the penumbral blur, the effect of the grating bridges as well as the detector point-spread function of ∼1.5 µm (r.m.s.). **a** One full Talbot-distance of the Talbot-carpet is depicted for two simulated grating periods. **b** Variance perpendicular to the propagation direction. Increased variances at larger propagation distances compared to Supplementary Figure 1 are due to the normalization to the maximum variance. **c**-**f** show transverse intensity profiles at the same location within the Talbot-carpet as Figure 1. Even for a fully coherent beam the amplitude of the oscillation is reduced to ∼0.3 and the intensity enhancement to ∼1.1.

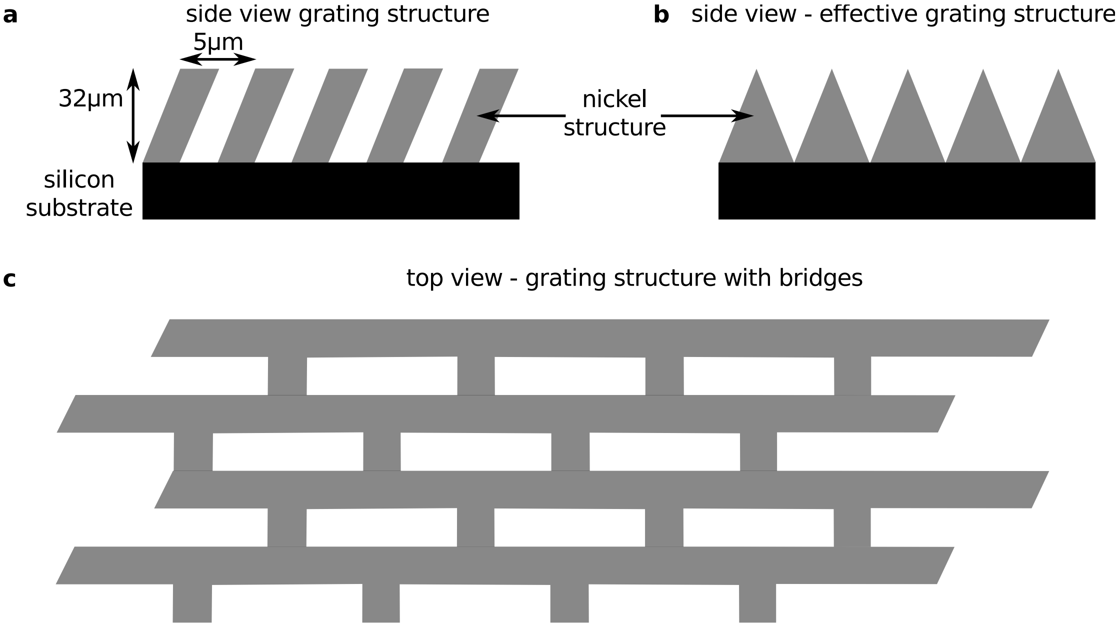

Supplementary Figure 4: Sketch of the grating structure used in the experiment. **a** Side-view of the real structure of the grating. **b** The resulting effective trigonal structure generated by such a grating. **c** Top view of the grating structure visualizing the bridges.

Supplementary Note 2: Performance Calculations

Comparison to a STXM

For a pencil beam (STXM-style) scanning at the same resolution, we assume the following parameters: A KB-mirror system with a typical transmission $T_{\mathrm{STXM}} = 0.9$ which is capable of focussing the full X-ray beam ($A_{\mathrm{beam}} = 5.6\,\mathrm{mm}\,\mathrm{x}\,3\mathrm{mm}$ in our case) into the same focal spot size ($A_{\mathrm{focus}} = 0.7\,\mu\mathrm{m}\,\mathrm{x}\,0.7\,\mu\mathrm{m}$) as the gratings. The incoming intensity I_0 is assumed to be the same for the scanning microscope as well as for the structured illumination. The STXM creates a flux enhancement $\mathrm{FE}_{\mathrm{STXM}} = \frac{A_{\mathrm{beam}}}{A_{\mathrm{focus}}} T_{\mathrm{STXM}} I_0 = 30.9 \cdot 10^6 I_0$ For the structured illumination, we assume a 2D-grating with 5 µm period and a detector with an ideal pixel pitch of 5 µm. Including the absorption of the grating, the transmission of the grating into multiple side-by-side foci is $T_{\mathrm{SI}} = 0.528$. Instead of the full beam size, only the intensity of the area of a single focus ($A_{\text{single-focus}} = 5\,\mu\mathrm{m}$ x $5\,\mu\mathrm{m}$) is focused into A_{focus}. Therefore, the flux enhancement for a single focus is $\mathrm{FE}_{\mathrm{SI}} = \frac{A_{\text{single-focus}}}{A_{\mathrm{focus}}} T_{\mathrm{SI}} I_0 = 26.9 I_0$. Accordingly, the acquisition time per point for the scanning approach is reduced to $t_{\mathrm{aq,STXM}} = \frac{\mathrm{FE}_{\mathrm{SI}}}{\mathrm{FE}_{\mathrm{STXM}}} t_{\mathrm{aq,SI}}$ for delivering the same flux at one focus. Thus, the STXM could acquire $N_{\mathrm{STXM}} = \frac{\mathrm{FE}_{\mathrm{STXM}}}{\mathrm{FE}_{\mathrm{SI}}} = 1.15 \cdot 10^6$ points during one acquisition using the structured illumination. In the latter case, $N_{\mathrm{SI}} = \frac{A_{\mathrm{beam}}}{A_{\mathrm{focus}}}$ points are acquired in parallel, where A_{beam} is the area of the beam that is covered by the detector. With $A_{\mathrm{beam}} = 5.6\,\mathrm{mm}\,\mathrm{x}\,3\,\mathrm{mm}$ in our case, we have $N_{\mathrm{SI}} = 0.67 \cdot 10^6$. The STXM, without any overhead, could acquire a factor $\frac{N_{\mathrm{STXM}}}{N_{\mathrm{SI}}} = \frac{T_{\mathrm{STXM}}}{T_{\mathrm{SI}}} = 1.7$ more points with the same fluence in the same time, which would be in favor of the scanning approach, but only if a KB-mirror system is used. In case that Fresnel zone plates are used, our approach would be faster due to the much lower efficiency of zone plates. Nevertheless, there is one significant drawback for a STXM. With an ideal detector resolution of 5 µm, a camera system with the same chip with 5120 x 3840 pixels could image a beam of 25.6 mm x 19.2 mm. Such beam sizes are readily available at inverse

Compton sources and too large for typical KB-mirror systems, thus requiring additional pre-focussing in the STXM approach. Furthermore, the speed required for data acquisition in the STXM approach would be extremely high. Our acquisition time was 300 ms per frame, which corresponds to a data acquisition time per illuminated spot of 262 ns in the scanning approach. As a result, the detector has to run at a frame rate of 4 MHz and the sample manipulation stages at a speed of 1910 mm/s during the scan, assuming a continuous scanning mode and neglecting time for de- and re-acceleration after each line. While the envisioned scan ranges are too large for fast piezo actuators, also direct drive stages can achieve this speed (e.g. Standa 8MTL220, Vilnius, Lithuania). However, de- and reacceleration of the stage after each line already takes ~0.2 s for the aforementioned stage. This corresponds to 1200 s for the 6000 lines required to scan the whole height of the field-of-view of 3 mm with 0.5 µm spacing. The total scan time would be $t_{\text{STXM}} = t_{\text{data-acq.,STXM}} + t_{\text{mot.mov.,STXM}} = 17.6\,\text{s} + 1200\,\text{s} = 1217.6\,\text{s}$ compared to $t_{\text{SI}} = t_{\text{data-acq.,SI}} + t_{\text{mot.mov.,SI}} = 30\,\text{s} + 2\,\text{s} = 32\,\text{s}$. Consequently, even the STXM employing a KB-mirror system would be slower due to the much larger amount of lines to be scanned compared to our approach, which requires only 10 lines.

Comparison to a TXM

Despite the fact that constructing a classical transmission microscope with a low magnification of 10 is not common, we consider such a hypothetical system here. Consider a hypothetical TXM with a KB-mirror system ($T_{\text{condensor}} = 0.9$) focussing the incoming beam (intensity I_0, $A_{\text{beam}} = 5.6\,\text{mm} \times 3\,\text{mm}$) down to $A_{\text{focus}} = 0.56\,\text{mm} \times 0.3\,\text{mm}$. In order to achieve the resolution, yet again the beam on the detector has to be $A_{\text{det}} = 5.6\,\text{mm} \times 3\,\text{mm}$. For good optical images KB-systems are not always the best solution, therefore compound refractive lenses with typical transmissions of $T_{\text{obj.lens}} = 0.4$ might be used as an objective lens. The intensity in the sample plane is $I_{\text{sam}} = T_{\text{condensor}} I_0 \frac{A_{\text{beam}}}{A_{\text{focus}}}$, the intensity on the detector plane is $I_{\text{det,TXM}} = T_{\text{obj.lens}} I_{\text{foc}} \frac{A_{\text{focus}}}{A_{\text{det}}} =$

$T_{\text{condensor}} T_{\text{obj.lens}} I_0 \frac{A_{\text{beam}}}{A_{\text{det}}}$. The total scan time is $t = n_{\text{images}} N_{\text{phot}}/F_{\text{pixel}}$, where N_{phot} is the number of photons per pixel required to form an image, $F_{\text{pixel}} = I_{\text{det}} A_{\text{px}}$ is the flux per pixel, A_{px} the detector pixel size and n_{images} is the number of images required to scan the object. If the same detector is used for both techniques, N_{phot} is the same for both cases, as well as A_{px}. If $\frac{t_{\text{TXM}}}{t_{\text{SI}}} > 1$ our approach is faster. The number of required images with our method is 100.

$$\frac{t_{\text{TXM}}}{t_{\text{SI}}} = \frac{\frac{N_{\text{phot}}}{F_{\text{pixel,TXM}}} n_{\text{images,TXM}}}{\frac{N_{\text{phot}}}{F_{\text{pixel,SI}}} n_{\text{images,SI}}} = \frac{F_{\text{pixel,SI}}}{F_{\text{pixel,TXM}} \cdot 100} n_{\text{images,TXM}} =$$

$$= \frac{T_{\text{SI}}}{T_{\text{condensor}} T_{\text{obj.lens}} \frac{A_{\text{beam}}}{A_{\text{det}}} 100} n_{\text{images,TXM}} > 1.$$

For the lenses and grating efficiency discussed before, this results in the condition $n_{\text{images,TXM}} > 68.2$ for our scanning technique to be faster. With $A_{\text{focus}} = 0.56\,\text{mm} \times 0.3\,\text{mm}$ the actual amount of scans is $n_{\text{images req.,TXM}} = \frac{A_{\text{beam}}}{A_{\text{focus}}} = 100$. Accordingly, our method would also outperform such a hypothetical TXM covering the same field-of-view with the same resolution.

References

[1] Wilde, F. *et al.* Micro-CT at the imaging beamline P05 at PETRA III. *AIP Conference Proceedings* **1741**, 030035 (2016).
